# Farm-level survey data on participation in agri-environmental schemes from regions in Germany, Czechia and England

**DOI:** 10.1016/j.dib.2026.112823

**Published:** 2026-05-06

**Authors:** Meike Will, Sofia Biffi, Emmanouil Tyllianakis, Tomáš Václavík, Birgit Müller

**Affiliations:** a Helmholtz Centre for Environmental Research - UFZ, Department of Ecological Modelling, Permoserstraße 15, Leipzig, 04318, Germany; b School of Geography, University of Leeds, Woodhouse Lane, Leeds, West Yorkshire LS2 9JT, United Kingdom; c Department of Agroecology, Aarhus University, Tjele, 8830, Denmark; d Sustainability Research Institute, University of Leeds, Woodhouse Lane, Leeds, West Yorkshire LS2 9JT, United Kingdom; e Institute of Energy and Sustainable Development, De Montfort University, Leicester, LE1 9BH, United Kingdom; f Department of Ecology and Environmental Sciences, Faculty of Science, Palacký University Olomouc, Šlechtitelů 241/27, Olomouc, 783 71, Czech Republic; g Global Change Research Institute of the Czech Academy of Sciences, Department of Climate Change Impacts on Agroecosystems, Bělidla 986/4, Brno, 603 00, Czech Republic; h Korea University, Department of Geography, 145 Anam-ro, Seoul, Seongbuk-gu 02841, Republic of Korea; i Modelling of Human-Environment-Systems, Brandenburg University of Technology (BTU) Cottbus-Senftenberg, Cottbus, 03046, Germany; j iDiv German Centre for Integrative Biodiversity Research Halle-Jena-Leipzig, Leipzig, Germany

**Keywords:** Agri-environment and climate schemes, Farmer behaviour, Sustainable agriculture, Discrete choice experiment, Policy design

## Abstract

This dataset contains survey data from 250 farmers located in Saxony (Germany), southern Moravia (Czech Republic), and England and their decisions on adopting agri-environmental schemes (AES). The online survey was conducted between September 2021 and April 2022 and included mostly closed-ended questions and a discrete choice experiment (DCE). The survey data covers farm characteristics, personal values and socio-demographic questions, as well as experiences with existing AES and factors influencing farmers’ participation in specific schemes. With the DCE, we collected preferences of farmers across case studies for a selection of contract characteristics. Respondents had to choose between four alternative AES and a “no scheme” option in which farmers would not receive any funding for agri-environmental practices. In addition to the offered payment level, contract length, bureaucratic effort and advisory support were key attributes that differed between the schemes.

Specifications Table

**Table:** 

Subject	Social Sciences
Specific subject area	*Farmer decision-making on adoption of agri-environmental schemes*
Type of data	Cleaned
Data collection	Data collection in the three case studies took place between September 2021 and April 2022. Data from farmers were collected in online questionnaires and were disseminated via established farmer networks from the co-authors in Germany and the Czech Republic, while in England all but nine questionnaires came from a country-wide farmer panel purchased from the survey company Qualtrics. The median completion time in the retained questionnaires was 21 min for Germany and Czech Republic and 9 min for England (experienced with farmer surveys) sample.
Data source location	Institution: Helmholtz Centre for Environmental Research, UFZ City/Town/Region: Saxony (Germany), southern Moravia (Czech Republic), England Country: Germany, Czech Republic, United Kingdom
Data accessibility	Repository name: Zenodo Data identification number: 10.5281/zenodo.17551096 Direct URL to data: 10.5281/zenodo.17551096
Related research article	E. Tyllianakis; M. Will; T. Václavík; G. Ziv, Drivers and Preferences of European Farmers for Agri-Environmental Public Goods Schemes: A Two-Stage Analysis, Journal for Nature Conservation, 86 (2025) 126,912. 10.1016/j.jnc.2025.126912 [1]

## 1 Value of the Data

• Agri-environmental schemes within the European Union’s Common Agricultural Policy aim to promote sustainable agricultural management; however, their adoption has been lower than expected. This data helps to understand the factors that influence farmers' participation in these schemes.
• The dataset facilitates a comparative analysis of the motivations behind adopting agri-environmental schemes in three European case studies across three regions: two geographically and culturally similar regions in Germany and the Czech Republic, and one region (England) that differs geographically, culturally, and politically from the other two.
• By analyzing the discrete choice experiment in combination with a detailed questionnaire, the effects of the characteristics of farms and farmers in conjunction with their personal attitudes can be used to gain insights for designing AES that are better tailored to the needs of farmers.
• Researchers and policy makers can draw conclusions from the data to design more effective policies and improve existing agri-environmental strategies by aligning them with farmers’ expectations.

## 2 Background

Agri-environmental schemes (AES) are intended to promote sustainable agriculture. However, under the European Union’s Common Agricultural Policy, adoption of AES in their current implementation has been lower than expected [2,3]. A better understanding of why farmers choose to participate in AES and why they may refrain from doing so is crucial for designing policies that meet farmers’ expectations and thus gain broader acceptance.

On this basis, we developed an online survey containing closed questions about the characteristics of the farms and farmers and their personal preferences, as well as a discrete choice experiment in which farmers had to express their preferences for AES. The survey was conducted in three case studies as part of a wider, multi-country European project [4]. Two regions in Germany (DE) and the Czech Republic (CZ) were chosen due to their geographical and cultural similarities, while England (EN) was selected as the third case study region due to its geographical distance and political differences. The two remaining case study regions of the project (in Serbia and Spain) were not included in the dataset due to incompatibilities in the survey structure (in the case of Serbia, which is an EU candidate country where currently no AES are implemented) or data limitations (in the case of Spain, where the survey was completed by an insufficient number of participants).

Our data set can help identify which features of AES design promote its adoption and which tend to hinder it. Since the reasons for and against participating in AES are captured in various ways within the survey (i.e., using an in-depth questionnaire and a DCE), the data can provide a nuanced picture of the decision-making process and thus help policy makers tailor the design of AES to farmers’ expectations. Comparing three countries can furthermore help to identify specific differences in the importance of individual features of an agri-environmental program but also highlight common priorities between countries. Part of the dataset presented here was previously used in a research article analysis on the drivers and preferences of European farmers for AES [1].

## 3 Data Description

Data collection took place between September 2021 and April 2022. The link to the online survey was distributed via established farmer networks and advertised on social media (e.g., Twitter, currently X) in local languages. In England, attempts to distribute the survey via social media and farmers’ associations resulted in a low response rate, with only nine usable responses received from the original study area (Humber region). Thus, the survey company Qualtrics was contracted to administer the survey and collect responses from a nationwide panel of farmers, thereby expanding the original case study area to the whole of England. As an incentive to participate in the survey, respondents were offered the chance to enter a raffle to win one of four vouchers for a local store worth €100 each in DE and CZ or one of four food hampers worth £100 each in EN.

**Fig. 1:**
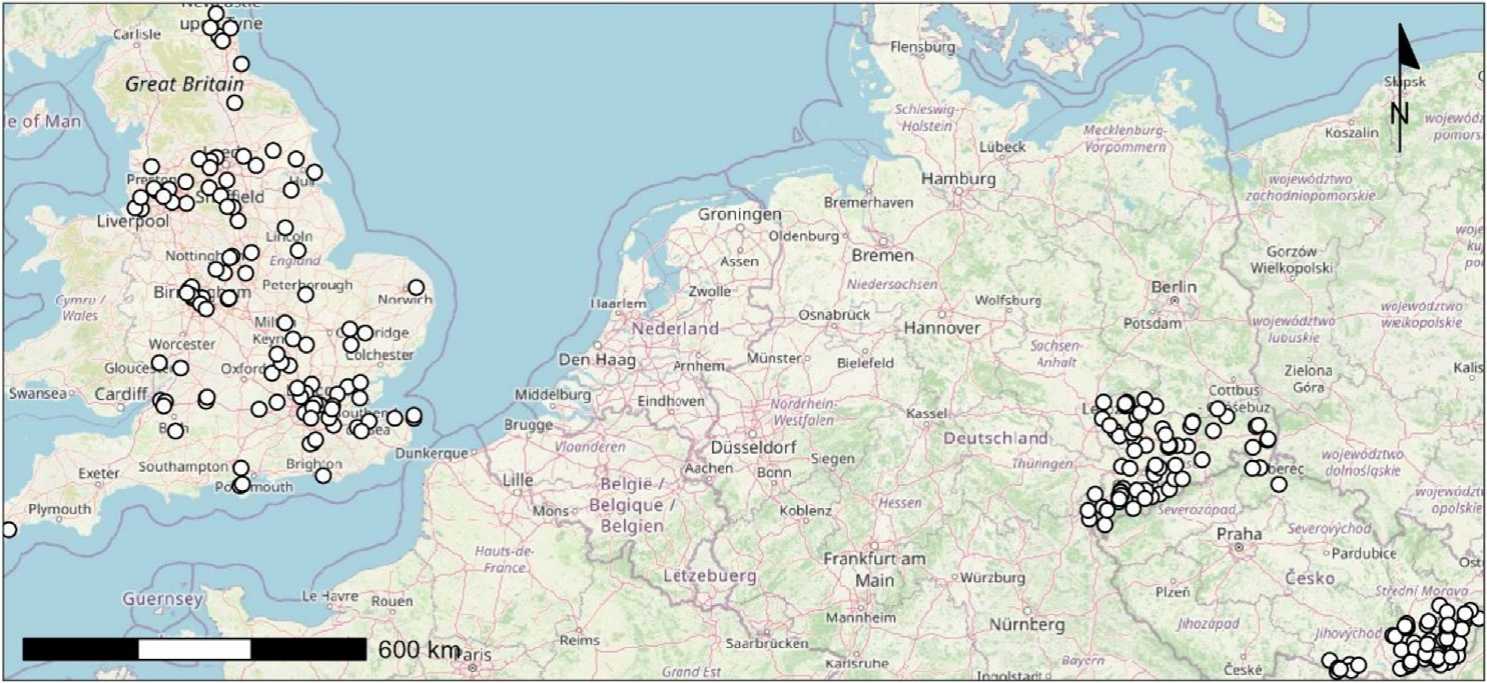
Geographical location of survey participants in the three case studies according to their postcodes. For some areas, several respondents participated.

A total of 440 questionnaires were collected (146 in DE, 140 in CZ, 154 in EN). After removing incomplete questionnaires, 250 responses remained (74 in DE, 69 in CZ, 107 in EN). Due to the small sample size, we, however, cannot guarantee a representative sample of the farming population either overall or for the individual case studies. The median completion time for the remaining questionnaires was 21 min for DE and CZ and 9 min for EN (mostly experienced with farmer surveys) sample. Fig. 1 shows the geographical distribution of the survey participants.

Depending on the questions, the dataset includes numerical, ordinal and free-text responses. Furthermore, questions with single- or multiple-choice options are covered. A csv file of the dataset after data cleaning (*Data_CZ_cleaned.csv; Data_DE_cleaned.csv; Data_EN_cleaned.csv*), a PDF of the survey in three languages (*Survey_CZ.pdf, Survey_DE.pdf, Survey_EN.pdf*) and the codebook with the description of the variables (Codebook_BESTMAP_Survey.xlsx) are available at Zenodo [5]. All data are in a format that does not allow tracing them back to individual respondents.

## 4 Experimental Design, Materials and Methods

The survey was designed and administered using the survey platform Qualtrics. We pretested the questionnaire design and the DCE with a small sample of farmers from the three case studies.

The questionnaire consisted of five sections. It started with an explanation of the project and its objectives, as well as a brief overview of the content of the survey. Participants were informed about the expected duration of the survey (20 min), ethical approval (in EN and CZ), as well as confidentiality, data use, and anonymity. Further, we informed them that they could request the deletion of their answers up to 30 days after completing the survey. By continuing to the next page of the survey, farmers confirmed their informed consent to participate in the survey.

**(1) Information on farm characteristics**

The first section of the survey focused on collecting information on farm characteristics (Table 1). This included questions about location, farm type, land use and management practices, and advisory support. The survey followed a hierarchical structure, with some question being presented only if relevant to previous answers. Specifically, questions on arable crops were only presented if the respondent indicated that they managed arable land, while questions about livestock were conditional on reporting either land or permanent grassland, with an additional option for improved grassland in EN. The EN survey also collected information on area under long-term lease and area of common land, which are not typical for DE and CZ.

**Table 1:** Information on farm characteristics collected in the survey.

Characteristics	Description	Type / Scale of response
**Location**	Postcode of farm (in EN only first half of postcode for privacy reasons)	Free text
**Broad classification**	Type of farm; conventional/organic status; participation in quality programmes or voluntary certification schemes	Multiple choice
**Land use and land ownership**	Area managed for different land types; rented area	Numeric
**Farm dispersion**	Share of land >3 km away from farmstead	6-point Likert scale
**Arable crops**	Top five most economically important crops in the last year	Free text
**Livestock**	Presence of livestock; top five most important livestock types and number of animals	Free text
**Perceived soil quality**	Compare average fertility of farm soil to district average	5-point Likert scale
**Advisory support**	Frequency of consultations with agricultural advisory/extension services: general, nature conservation, AES-specific	5-point Likert scale

**(2) Discrete choice experiment**

The second section of the questionnaire contained the DCE. In the choice experiment, farmers were given the option to choose between four hypothetical AES (i.e., flower strips/areas, cover crops, maintaining permanent grassland, and conversion of arable land to permanent grassland). The first three AES were chosen because they were offered as AES practices with largely similar implementations and because they are widely adopted in all regions. Conversion of arable to permanent grassland did not meet these criteria, as it is not offered in all regions and its adoption is limited, but it was included due to its relevance for future AES implementations under the EU’s Common Agricultural Policy. Participants are presented with definitions of the hypothetical schemes in relation to existing contract characteristics across case studies.

The characteristics of these contracts (Table 2), which were included as attributes in the DCE, are contract duration (1, 5 and 10 years), the possibility of advisory support during the contract period (yes or no), and the administrative effort for the farmer (low, medium and high). The compensation per hectare enrolled in the proposed contract per year is the final contract characteristic, with five different levels for each country. A complete list of payment levels for all schemes and countries is provided in Table 3. The reference levels for each flower strips, cover crops, and maintaining grassland were based on official figures for such payments in CZ, DE, and EN, while reference levels for conversion from arable to grassland were informed by the pre-test phase. The remaining compensation levels consisted of a reduction of approximate 25 %, a reduction of 15 %, an increase of 15 %, and an increase of 25 % compared to the reference level. As a fifth option, participants were offered an opt-out option, whereby they would not receive any compensation for the AES carried out on their land.

**Table 2:** Attributes and corresponding levels in the DCE. Levels in bold indicate the reference level.

Attribute	Levels
Contract duration	1; **5**; 10 years
In-person advisory support	Yes, free of charge; **No**
Administrative effort	Low; **Medium**; High
Yearly payment	5 payment levels for each of the four contract types (see Table 2 for the full list for each country)

**Table 3:** List of payment levels for each country and AES in the DCE with sources used as reference.

AES	Payment levels				
**CZ (EUR/ha/year)** [6]					
Flower areas/strips	450	525	600	675	750
Cover crops	65	75	85	95	105
Maintaining grassland	140	165	190	215	240
Conversion of arable land to grassland	900	1050	1200	1350	1500
**DE (EUR/ha/year)** [7]					
Flower areas/strips	630	735	840	945	1050
Cover crops	65	75	85	95	105
Maintaining grassland	240	285	330	375	420
Conversion of arable land to grassland	1200	1400	1600	1800	2000
**EN (£/ha/year)** [8]					
Flower areas/strips	410	550	620	690	825
Cover crops	90	120	135	150	180
Maintaining grassland	140	190	215	240	285
Conversion of arable land to grassland	880	1100	1250	1400	1665

**Fig. 2:**
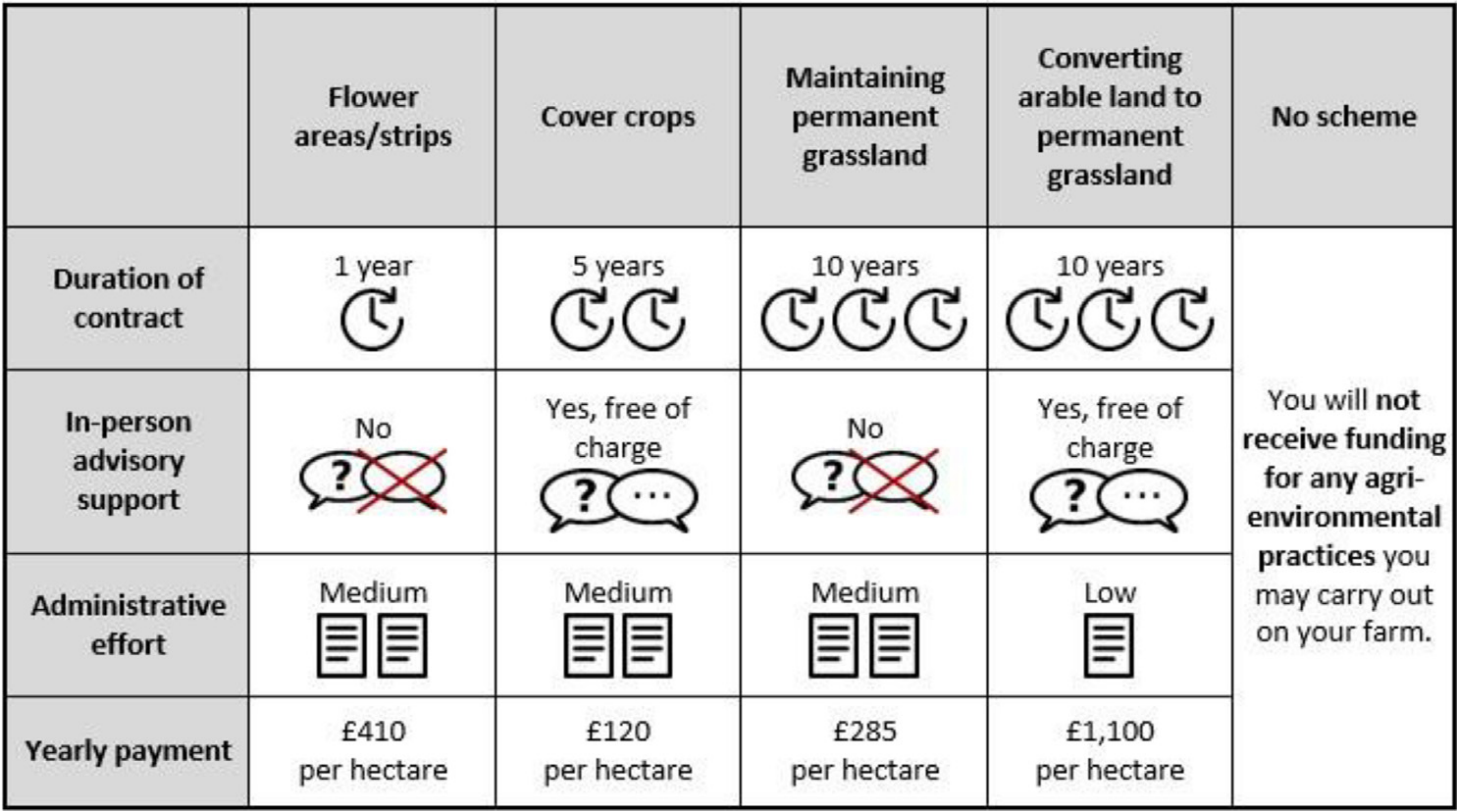
Example of a choice card for the EN case study.

In line with the usual split sample comparison approach (e.g., [9]), half of the participants were presented with the opt-out option in random order, while the other half were presented with the options in such a way that the opt-out option was always at the end (as shown in Fig. 2). This was done to investigate possible ordering effects with regard to the position of the opt-out option (referred to in the literature as “position order effects”) [10,11,12].

The framing of the DCE is tailored to the framing of AES with which participants can already be familiar (e.g., the contracts in EN are presented as contracts under the Countryside Stewardship), stressing the hypothetical nature of the options. A ‘cheap talk’ script, i.e., a script that informs participants that their responses in a hypothetical scenario may differ from their actual choices in real life, is used to incentivize participants to answer truthfully [13]. Before each choice, participants are reminded the following: “If you do not have permanent grassland, please do not consider the option “Maintaining permanent grassland”” to ensure that the options selected are realistic. After each choice except for the “no scheme” option, respondents are asked to indicate the percentage of land they would be willing to enrol in the contract they have just selected, both in terms of arable land and of grassland. If respondents select the “no scheme” option every time they are asked to choose reasons from the following options: “I am generally not willing to enrol in agri-environmental schemes”, “I did not find the options suitable for my land or current situation”, “Enrolling in such schemes would be a bad financial decision for me” and “There is no need for actions to protect the environment in my farm”. A question was also added as to why the participant has never selected the option ‘conversion of arable land to grassland’. Here, the same options were offered as for those who decided against participation (opt-out) each time, in order to determine why this hypothetical scheme is not feasible.

To minimize the sample size required to estimate the actual preferences of farmers in the three case studies, we created an S-efficient statistical design [14] for a labelled choice experiment with two blocks and six choice cards each via Ngene (ChoiceMetrics, 2012, Version 1.1.1.) with fixed priors. Priors for contract duration and administrative effort were derived from the pooled estimates of [15], and advisory support priors were obtained from [16]. This design was pretested with 13 completed questionnaires from the three case studies, and estimated coefficients were used as priors to improve the statistical design from an S-error of 70.62 to the final value of 60.02 (D-error 0.0047 and 0.0045 accordingly). All countries share the same statistical design.

**(3) Prior experience with AES**

**Fig. 3:**
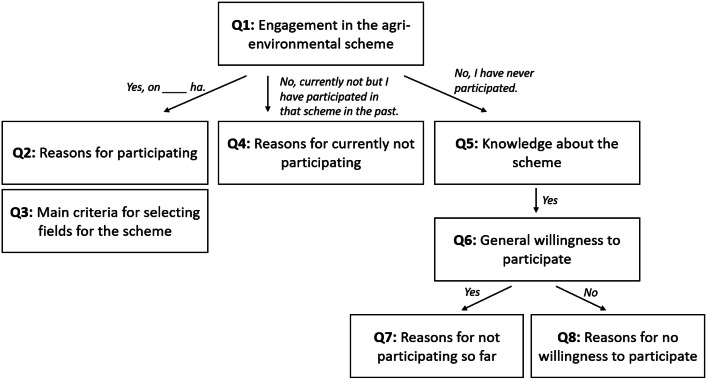
Selection of follow-up questions on prior experience with a specific AES depending on current and previous participation in that scheme.

The **third section** focused on prior experiences with AES which has been shown to increase farmer participation [17,18,19]. In each case study, three existing schemes were selected that most closely matched the hypothetical schemes presented in the DCE (see Table 4). For each scheme, participants had to indicate whether they (1) currently participate in it (if so, on how many hectares), (2) do not currently participate in it but have participated in that scheme in the past, or (3) have never participated in it. Depending on their answer, farmers were directed to different follow-up questions about the reasons for their (not) participation (see Fig. 3 for a graphical summary of the selection of questions). For cover crops, we additionally asked about the main crop that the farmer would cultivate or did cultivate after the cover crops.

**Table 4:** AES selected in the three case studies (description in local language and English translation).

Case study	Selected AES		
**CZ**	Biopásy (flower strips)	Ošetřování travních porostů (grassland maintenance)	Zatravňování orné půdy (Grassland conversion of arable land)
**DE**	Mehrjährige Blühflächen oder einjährige Blühflächen (perennial flowering areas and annual flowering areas)	Anbau von Zwischenfrüchten (cultivation of cover crops)	Spezielle artenschutzgerechte Grünlandnutzung (Special species-appropriate grassland use)
**EN**	Flower-rich margins and plots	Winter cover crops	Management of species-rich grassland

**(4) Personal views of farmers**

Section four of the survey dealt with personal views of the participants. In particular, participants were asked to rate their agreement with six statements on a 5-point Likert scale. The statements investigated the participants’ agreement with (1) food production as the sole objective of farming, (2) the importance of safeguarding the environment, (3) profit maximization as an important objective of farm management, (4) society’s perception of their farm as environmentally friendly, (5) interaction with other farmers, and (6) the application of new technologies on their farm.

For EN, an additional section was added focusing on the Sustainable Farming Incentives scheme, which at the time of the survey was intended to gradually replace the existing payment system [20]. In this section, farmers were asked to rate six statements about how they would react if they no longer received Basic Payments (i.e., UK's main farm subsidy at the time) on a 5-point Likert scale.

**(5) Socio-demographic characteristics**

The last section of the questionnaire included questions on key socio-demographic characteristics. Specifically, we asked how many years the participant has been working in agriculture, the highest level of farming-related education that they had completed, and the share of their produce sold directly to consumers. Participants from full-time and part-time private farms were asked whether they already have a designated successor, whether they have other income sources besides farming, and if so, which share of household income is generated by activities other than farming. Finally, we asked about gender and age.

## Limitations

One limitation of the dataset is that the DCE deals with hypothetical schemes, while the rest of the questionnaire (especially section three on prior experience with AES) refers to actual measures available in the case study area. Although we aligned the hypothetical schemes with existing ones as much as possible, there were naturally some deviations, as we wanted to offer the same hypothetical schemes in all case studies. In addition, the data quality of the Qualtrics panel (EN case study) was lower than in the other two case studies, where we reached participants through local farmers' associations. Although Qualtrics participants had to indicate that they were farmers, we were unable to verify this information with the same reliability as the information provided by respondents in DE and CZ. In addition, we found that the median completion time for EN respondents was well below that of DE and CZ (21 minutes for DE and CZ and 9 minutes for EN). This could be because the population in which Qualitrics conducted the survey had already been exposed to online surveys, but it could also indicate that less attention was paid to the survey and less importance was attached to it. Overall, none of the three case studies included a sufficient number of participants to ensure a representative sample of the farmer population. Nevertheless, the survey provides insights into factors that influence farmers' decisions regarding AES, and the data analysis allows correlations to be drawn between farm and socio-economic characteristics, as well as personal views, and land use decisions.

## Ethics Statement

All participants involved in the study provided their written, informed consent to participate. Participation was voluntary and could be withdrawn at any time. Participants remained anonymous and their responses were dealt with in confidence. Ethical approval was obtained from the ethical commission at University of Leeds (application LTGEOG-057) and Palacký University in Olomouc.

## CRediT Author Statement

**Meike Will:** Conceptualization, Methodology, Data Curation, Investigation, Writing - Original Draft; **Sofia Biffi:** Conceptualization, Investigation, Writing - Review & Editing; **Emmanouil Tyllianakis:** Conceptualization, Methodology, Investigation, Writing - Review & Editing; **Tomáš Václavík:** Conceptualization, Investigation, Writing - Review & Editing; **Birgit Müller:** Conceptualization, Writing - Review & Editing.

## Data Availability

ZenodoFarm-level survey data on participation in agri-environmental schemes from regions in Germany, Czechia and England (Original data).
